# A survey of diagnosis and therapy of inborn errors of immunity among practice-based physicians and clinic-based pneumologists and hemato-oncologists

**DOI:** 10.3389/fimmu.2025.1597635

**Published:** 2025-06-05

**Authors:** Thomas Lehrnbecher, Alexandra Russo, Gernot Rohde

**Affiliations:** ^1^ Department of Pediatrics, Division of Hematology, Oncology and Hemostaseology, Johann Wolfgang Goethe University, Frankfurt/Main, Germany; ^2^ Department of Pediatric Hematology/Oncology, Center for Pediatric and Adolescent Medicine, University Medical Center, Johannes Gutenberg-University, Mainz, Germany; ^3^ Department of Pneumology, Clinic of Pneumology, Intensive Care Medicine and Sleep Medicine, University Medical Center, Justus Liebig-University, Marburg, Germany

**Keywords:** IEI, PID, APDs, immunodeficiency, survey, diagnostic landscape, treatment landscape, health services research

## Introduction

### Demographic aspects of IEI and overview

Inborn errors of immunity (IEI), formerly known as primary immunodeficiencies (PID), are a group of hereditary conditions that affect the functioning of the immune system. The number of identified IEIs currently amounts to a reported 559, and with continued genetic research and given the rapid pace of discovery it is expected to increase continuously (1). IEI may clinically manifest as unusually frequent or severe infectious complications (immunodeficiency), as autoimmunity or autoinflammation (immune dysregulation), and may be associated with syndromic multi-organ diseases or developmental disorders (e.g., ataxia teleangiectatica (AT)). Due to improved diagnostic procedures and reporting, it becomes increasingly clear that the actual prevalence is far higher than originally assumed (2, 3). According to some reports, between one out of 1200 and 2000 people could be affected (2), which would similar to the prevalence of multiple sclerosis (MS) (4). Latest studies suggest that there is a large number of genetic variants that in addition to other organ systems also affect the immune system; the incomplete penetrance in some cases makes it even more difficult to estimate the number of people affected, but the rate could be as high as 1:500 (5). Unfortunately, awareness for IEI compared to MS is disproportionately low among health care providers (HCPs) (6), which is partly due to the fact that this highly heterogeneous group of diseases often manifests in unspecific ways. For most patients with IEI, the first contact point will be a practice-based pediatrician or a general practitioner, but a significant proportion of patients will eventually present to a practice-based specialist or a pneumology or hemato-oncology department, as many IEIs manifest by recurring respiratory complaints, suspected lymphoproliferative disorder or blood abnormalities (7).

### Etiology and clinical spectrum of IEIs

The group of IEIs includes a large number of mostly hereditary diseases, though genetic mosaicisms or somatic mutations have also been described for a small number of conditions (8). Disease-causing variants can be located in genes whose function is predominantly related to the host immune defense, such as *STAT3* in the Hyper-IgE syndrome (9), or in genes with very fundamental functions, e.g. *ATM* in the case of ataxia telangiectasia (10). From the evolution of the terms PID and IEI, it can be seen that these diseases were initially thought to only affect immune defense, whereas more recently it has been recognized that they can manifest both by immune deficiency and immune dysregulation (11, 12). Some IEIs are accompanied by pathological lymphoproliferation, which can increase the risk for hematologic malignancy, in particular of lymphomas (13), but also poses a challenge to differential diagnosis (14). The International Union of Immunological Societies (IUIS) currently recognizes 10 classes of IEI, including combined immunodeficiencies (with or without syndromic features), predominantly antibody deficiencies or diseases of immune dysregulation (1).

Due to the variety of clinical manifestations, IEI patients may primarily present at different specialists, e.g., to the pneumologist with recurrent respiratory tract infections, bronchiectasis or autoimmune granulomas of the lungs, or to the hemato-oncologist with cytopenia, lymphoproliferation or lymphomas (15–17).

### Diagnosis and management of IEI patients

As is the case with many rare diseases, diagnosing an IEI patient may be a challenging task. As a helpful guide, various lists of warning signs and guidelines have been developed for the detection of IEI, including the ELVIS/GARFIELD criteria (12, 18–20). In case of suspected IEI, patients first undergo basic immunologic testing, the assessment of lymphocyte subsets and antibody subclasses, which may trigger genetic testing. Depending on the basic immunologic testing, only a fixed set of genes is usually analyzed from the dataset (virtual panel analysis), whole exome and whole genome sequencing are on their way of becoming standard of care.

After the diagnosis has been established, treatment depends on the specific IEI and may include several strategies (**Table 1**). This includes antibiotic prophylaxis and IgG substitution therapy (21–23) in immunodeficiency, whereas patients with disorders of immune regulation (autoimmunity, autoinflammation, lymphoproliferation) may benefit from treatment with immunosuppressants (24). In recent years, the elucidation of genetic causes has made it possible to offer targeted therapies for several IEIs. In some cases, approved drugs can be repurposed, but new drugs have been developed as well. Some examples include STAT inhibitors which can be used to treat different hyperinflammatory conditions or leniolisib, a PI3Kδ inhibitor currently approved in the US for patients with Activated PI3Kδ syndrome (APDS) (25–27). In some patients, hematopoietic cell transplantation (HCT) is indicated. Although it is associated with considerable risks, it can offer a real chance for cure to selected patients (28, 29). Newer strategies include gene therapy, e.g. for Chronic Granulomatous disease (CGD) (30) or Activated PI3Kδ syndrome (31), where patient stem cells are genetically altered to correct the disease causing variant (32, 33), but gene therapies are not widely available yet and their long term risks are insufficiently explored.

**Table 1:** Therapy options in IEI.

Mode of therapy	Treatments (examples)	Indications (examples)
Symptom oriented	Antibiotic prophylaxis Immunoglobulin replacement therapy	All IEIs with immunodeficiency (21–23)
Immunosuppression	Glucocorticoids Rapamycin Rituximab	IEIs with immune dysregulation (24)
Targeted	Abatacept	CTLA4 insufficiency (34)
	Leniolisib	APDS (27, 35)
Curative	Hematopoietic cell transplantation (28, 29)	SCID (36) CGD (37)
	Gene therapy (32)	APDS (31)

APDS, Activated PI3Kδ syndrome; CTLA4, cytotoxic T-lymphocyte-associated Protein 4; CGD, chronic granulomatous disease; SCID, severe combined immunoinsufficiency.

### Purpose of this study

The aim of the study was to get an overview of the IEI diagnostic landscape outside specialized IEI centers, in particular in practices and clinics that do not deal with IEI patients on a daily basis. The targeted physicians include office-based physicians, and in the second instance physicians from pneumology and hemato-oncology clinics. Key points we investigated were: Significance of the topic IEI day-to-day, awareness for signs and symptoms of IEI, number of suspected, diagnosed and referred or treated IEI patients, handling and management of IEI patients (tests performed in-house, patient referral, collaboration with IEI clinics/centers), request for training events and materials. Further, we compared the situation in clinics and doctor’s offices according to these criteria to identify potential issues in the diagnosis of IEI patients and to identify possibilities for improvement in recognition and consecutive diagnosis if IEIs.

## Results and discussion

### Participating centers and physicians

#### Telephone-based interviews of practices

**Figure 1:**
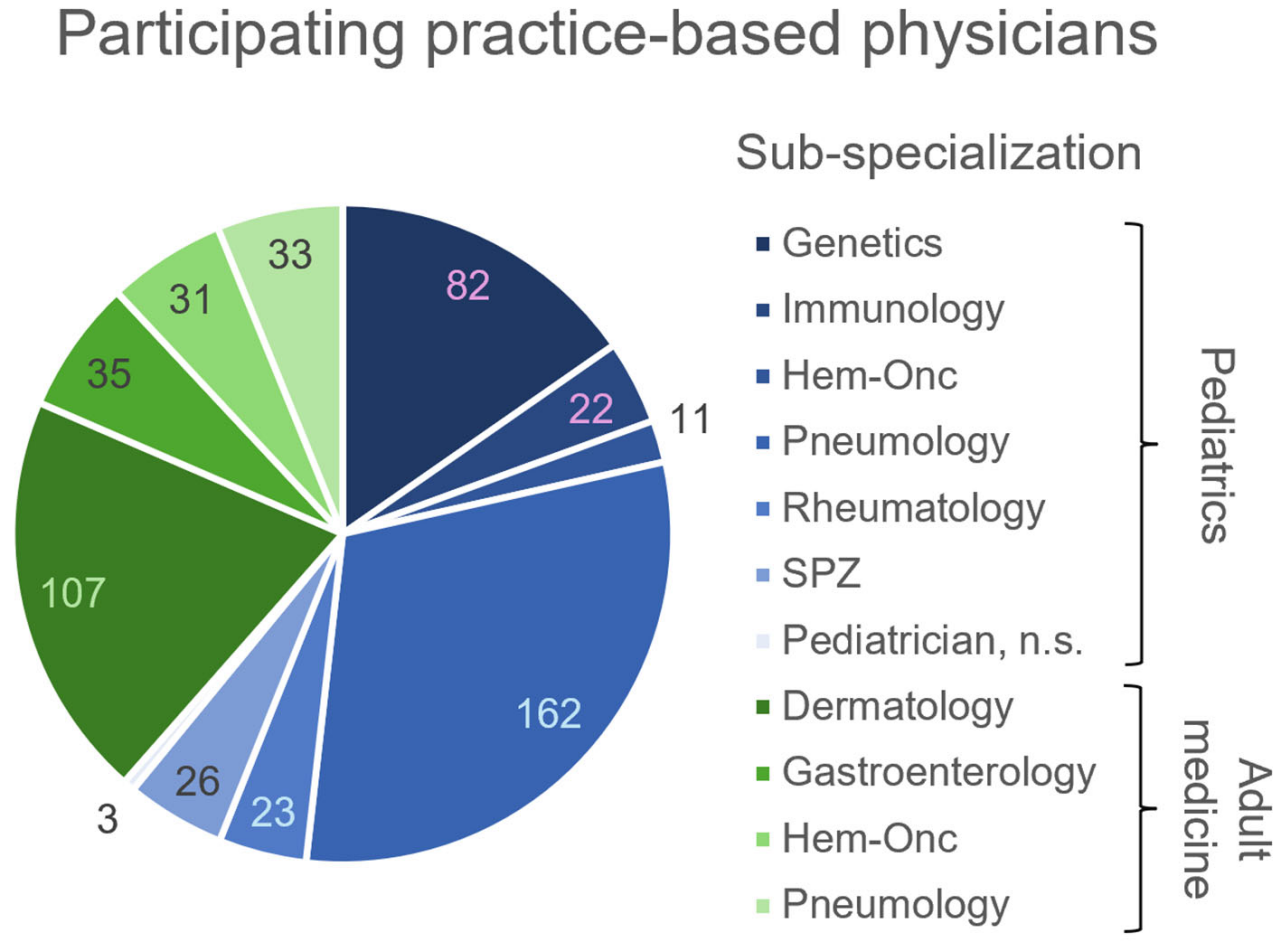
Number of practice-based physicians participating in the telephone survey (clockwise, n) per sub-specialization. Pediatrician sub-specializations indicated in blue, adult medicine sub-specializations indicated in green. n.s., non-specified; SPZ, social pediatric center.

**Figure 2:**
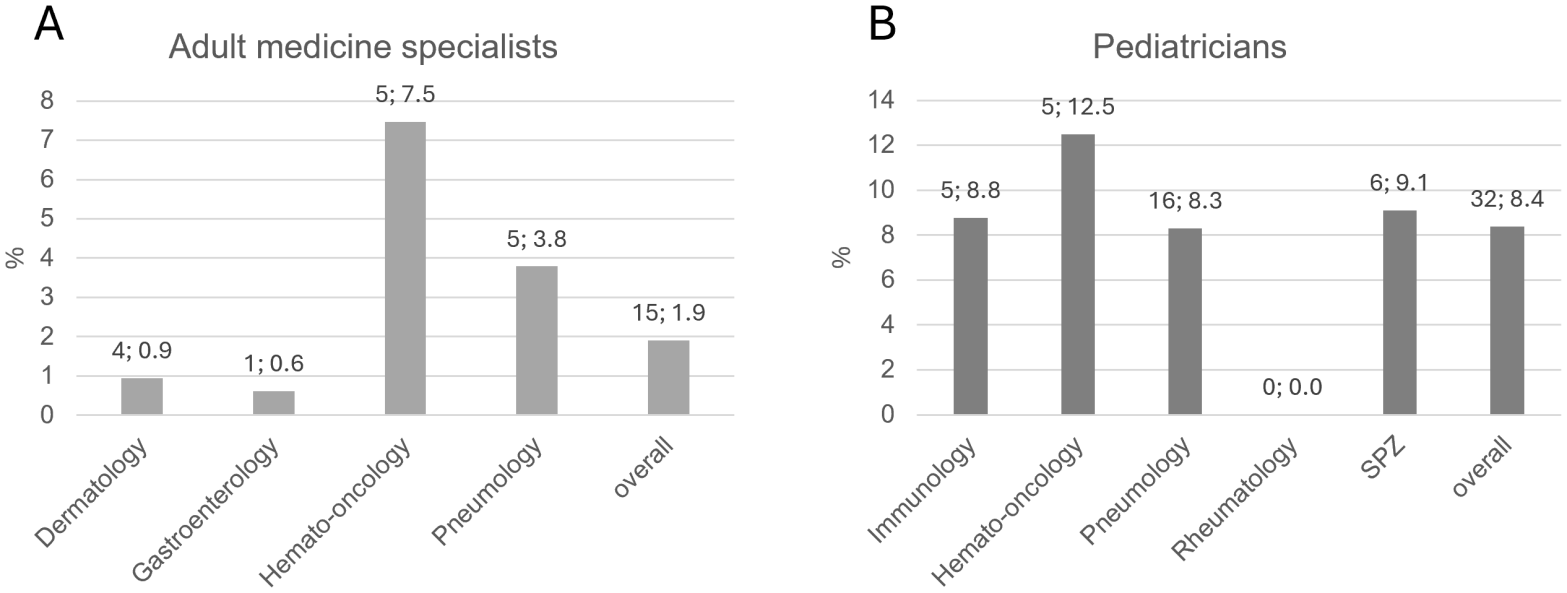
Number and proportion of practice-based **(A)** adult medicine specialists and **(B)** pediatricians per sub-specialization who have treated or currently treat IEI patients. Bars indicate percentages. Numbers above bar: absolute number; percentage. SPZ, social pediatric center.

The telephone survey of practice-based physicians reached out to a total of 1523 physicians in adult medicine and 1197 pediatricians, of which 206 and 329 were interviewed (**Figure 1**). The rate of positive responses was higher for pediatricians (329/1197 = 27.5%) than for specialists in adult medicine (206/1523 = 13.5%), suggesting a higher interest in the topic among pediatricians. This is not surprising, as IEI were considered for a long time (and still are) a domain of pediatric medicine. In order to enhance the pool of analyzable data information to selected questions was gathered from non-medical practice employees, for example on whether IEI patients had been treated before in the practice. Therefore, the total number of practices surveyed was higher than the number of surveyed physicians. Number and proportion of adult medicine specialists and pediatricians per sub-specialization who have treated or currently treat IEI patients are displayed in **Figure 2**. Overall, 1197 adult practices and 843 pediatric practices were reached.

#### Questionnaire and video interviews of clinic-based pneumologists and hemato-oncologists

**Figure 3:**
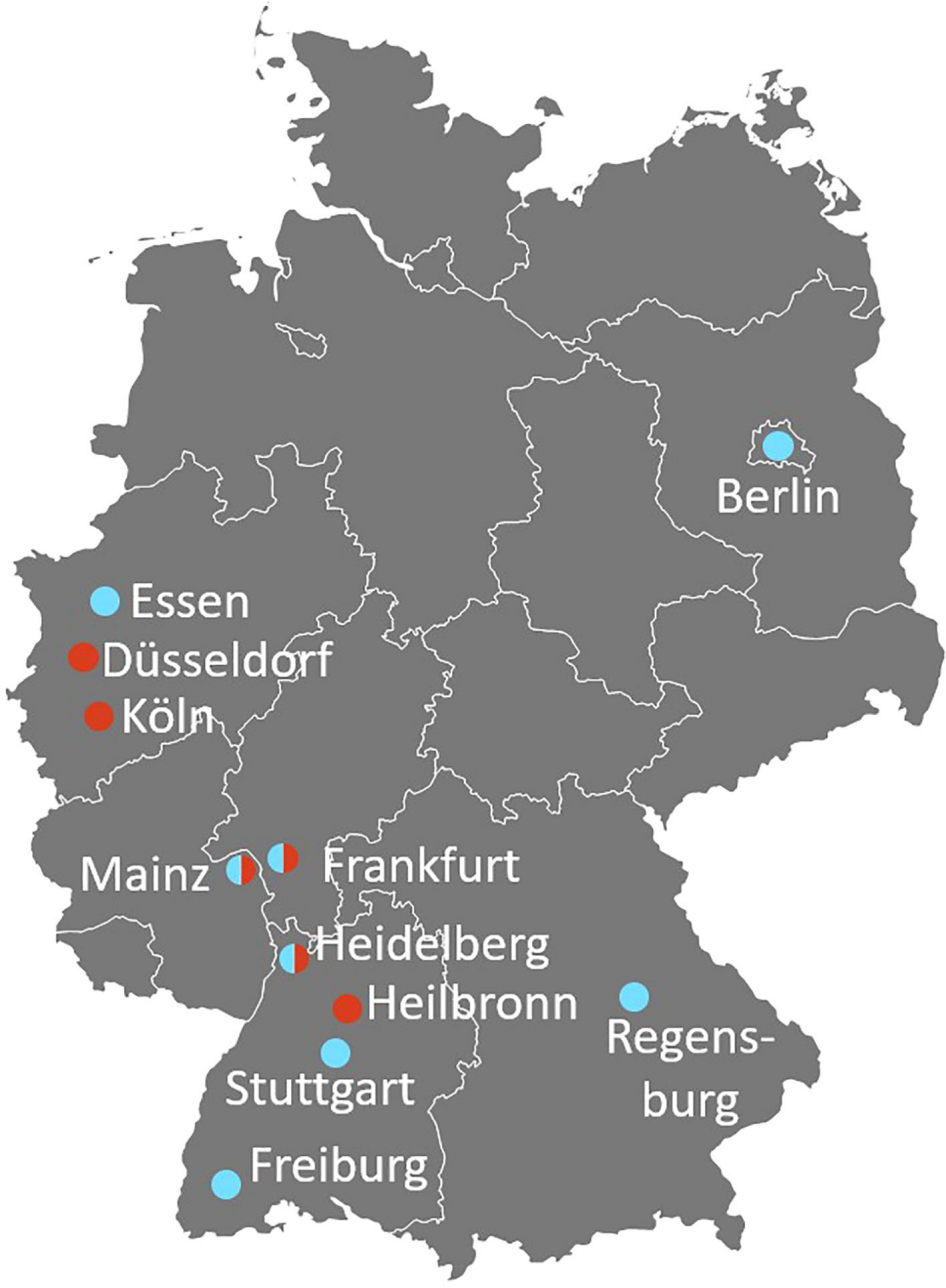
Geographic distribution of the participating clinic-based pneumologists (blue) and hemato-oncologists (red). Split bubble indicates that both, a pneumologist and a hemato-oncologist from the same city (not necessarily the same hospital) have been interviewed (survey with questionnaires + video interviews).

For the survey of clinic-based hemato-oncologists and pneumologists, 197 physicians from 69 different clinics and 5 private offices in 43 German cities were contacted via e-mail and/or telephone. One-hundred-nineteen practiced adult medicine (68 in pneumology, 46 in hemato-oncology) and 80 pediatric medicine (24 with a pneumology and 55 with a hemato-oncology sub-specialization). Two indicated treating both adults and pediatric patients. Fourteen of the contacted physicians agreed to answer the questionnaire and to participate in an interview (8 pneumology, 6 hemato-oncology). The rate of participation was twice as high among pediatricians (8/80 = 10%) than specialists for adult medicine (6/119 = 5%). Most participants were located in the South-West of Germany (**Figure 3**) despite centers throughout Germany were contacted. In general, the participation rate was low, although contact and follow-up were made both by e-mail and telephone and financial compensation was offered. This might indicate that especially centers with a very high interest in the topic itself took part.

The 14 participants were employed at specialized pneumology or hemato-oncology centers. The numbers of available beds were between 8 and 29 for hemato-oncology and between 25 and 130 for pneumology. However, in many cases several departments shared some of the beds and different clinics had varying degrees of access to beds in intensive care units or day clinics.

**Figure 4:**
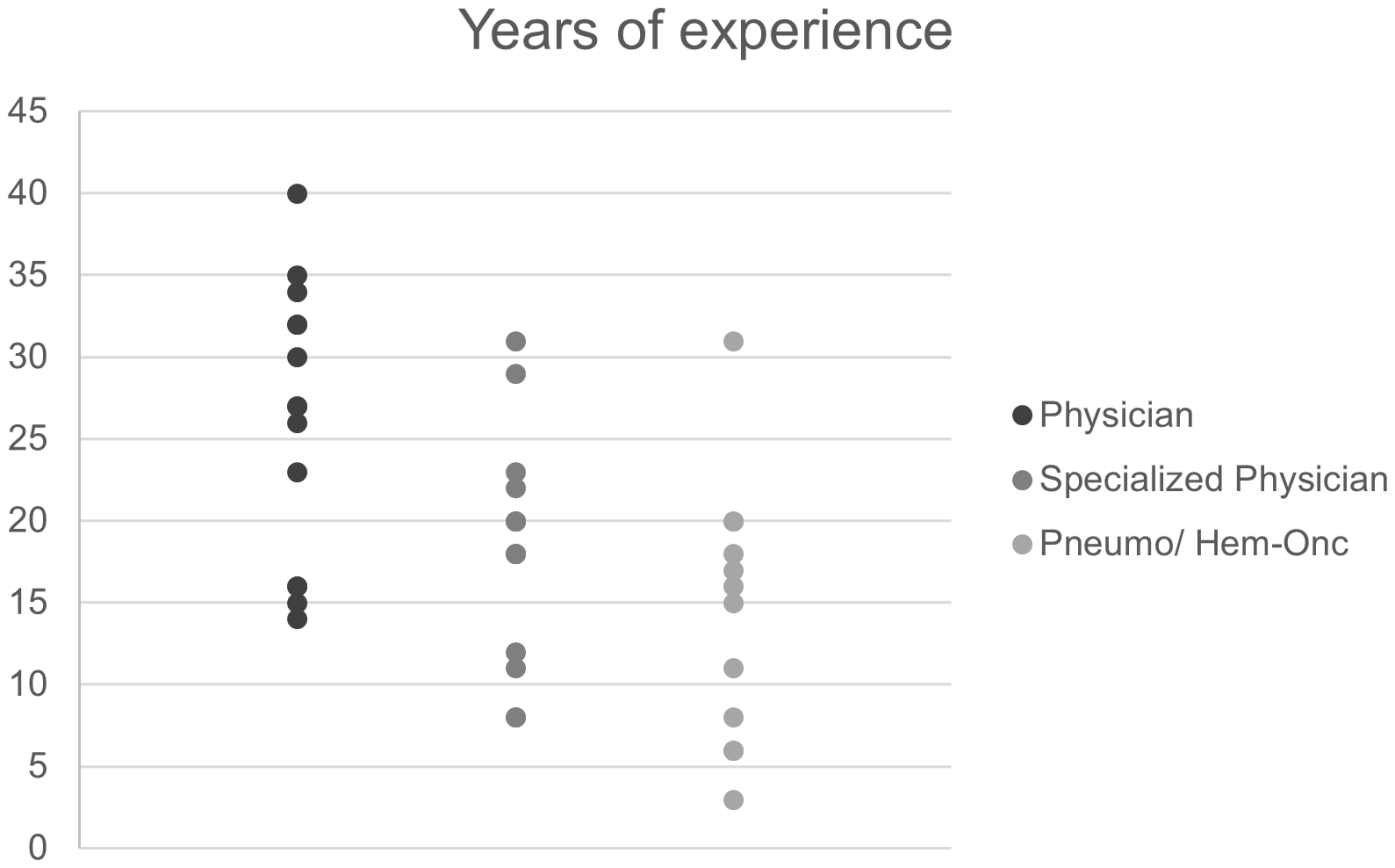
Work experience of clinic-based pneumologists and hemato-oncologists (interviewees). “Physician” refers to years since license to practice medicine and “Specialized physician” to years since attaining a specialization (e.g. internal medicine, pediatric medicine).

Interview partners had experience as physicians, specialized physicians and specialists for hemato-oncology or pneumology for a median of 27 years (range: 14 to 40), 20 years (range: 8 to 31) and 15.5 years (range: 3 to 31), respectively (**Figure 4**).

### Awareness of IEI and significance of IEI patient care in daily routine

#### Practice-based physicians

Among practice-based physicians, the treatment of IEI patients seemingly takes up little space in everyday practice. In total, physicians estimated the number of IEI patients ever treated at 19-31 (adult specialists) and at 45-70 (pediatricians). Relatively fewer adult specialists had treated IEI patients compared to pediatricians (1.9% vs 8.4%, **Figure 2**). Generally, pediatricians are more familiar with IEI warning signs than practitioners in adult medicine, although only 6 practices in total used the ELVIS/GARFIELD criteria. Unfortunately, it remained unclear in what circumstances they suspect IEI, which could be a focus of a future survey. Based on the presumed prevalence of IEI (2, 3) it can be speculated that there is a significant number of patients in whom a specific IEI is not correctly diagnosed and therefore do not receive the correct treatment. It is therefore crucial to sensitize more physicians to this disease spectrum.

#### Clinic-based pneumologists and hemato-oncologists

The general level of awareness for the existence of IEI patients was relatively high among all the interviewed clinic-based pneumologists and hemato-oncologists, although the number of treated patients varied significantly from clinic to clinic. All but one interview partner were aware of the ELVIS/GARFIELD criteria and were using them to identify suspected IEI cases. Nonetheless, the number of suspected, diagnosed and treated IEI patients was very low at clinics that were not specialized on IEI patients, with suspected cases in the low tens and confirmed cases of <1 to 5 per year. Not surprisingly, IEI care was considered a secondary topic which does not come up frequently in daily routine. In contrast, five interview partners (2/8 pneumologists, 3/6 hemato-oncologists) described their institutions as designated IEI clinics and reported significantly higher annual numbers of suspected IEI cases (50–150) and confirmed patients (15–150), respectively. This number also includes patients who were referred from other clinics (**Supplementary Tables S1** and **S2**).

The comparatively high level of expertise in the surveyed groups was also reflected by the fact that most participants had already taken part in several immunology training courses, some of them also as presenters. The low rate of participants relative to contacted persons bears the risk of self-selection towards physicians who are already sensitized to IEI which is also reflected by the fact that 5 out of 14 participants stated that they work at a clinic specializing in IEI. Therefore, only limited conclusions can be drawn about the average level of awareness of IEI in pneumology and hemato-oncology clinics.

### Diagnosis of IEI patients by clinic-based pneumologists and hemato-oncologists

Due to the wide range of symptoms and severity of IEI, we asked which symptoms were considered to be particularly indicative of IEI, including susceptibility to infection, abnormal laboratory findings, organ manifestations (e.g. bronchiectasis or lymphadenopathy) and developmental disorders or syndromic characteristics.

**Figure 5:**
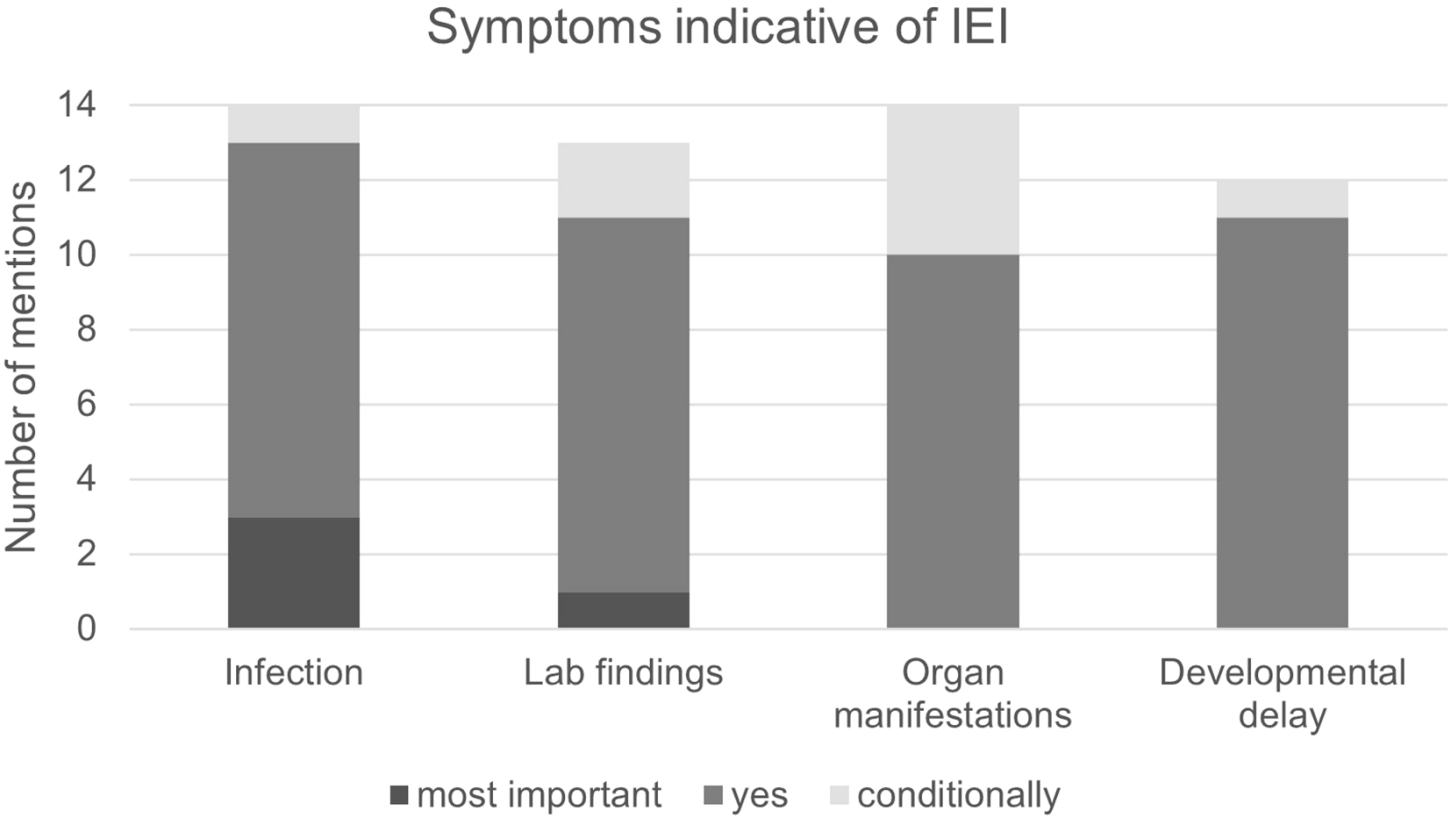
Symptoms indicative of IEI. Infection: High susceptibility to infection as per the ELVIS criteria. Lab findings: Abnormal findings in blood indicative of IEI. Organ manifestations: Abnormal findings in the organs that may indicate IEI, e.g. bronchiectasis or splenomegaly. Developmental delay: For example, short stature, facial anomalies or neurological developmental disorders. Most important: Sign can prompt a suspected IEI diagnosis on its own. Yes: Considered an important sign but not sufficient to prompt suspected IEI diagnosis on its own. Conditionally: Is a sign of IEI depending on its manifestation and in combination with other signs.

Most participants (10/14) stated that all parameters listed in the questionnaires could, in principle, be indicators of IEI, but that they are weighted differently (**Figure 5**). For example, bronchiectasis and lymphadenopathy can be indicators of IEI, but only lead to a suspected diagnosis in combination with other factors. In contrast, recurrent severe infections were considered the strongest single indicator. The same is true for developmental disorders such as delayed growth, hearing loss and facial dysmorphia, which also were considered conditional indicators of IEI. In case of susceptibility to infections, frequency of infections alone was reported to be insufficient to formulate a suspected diagnosis; instead, attention should be paid to unusual pathogens, e.g. fungal infections of the lungs. Other participants cited autoimmune phenomena (7/14 participants, specifically 2/8 pneumologists, 5/6 hemato-oncologists), e.g. autoimmune cytopenia, enteropathy and sarcoidosis.

These statements correspond well with the warning signs formulated by the professional societies (12, 19, 20) and indicate that the interviewees are largely well informed about IEI.

#### Bronchiectasis of unclear origin

One known complication of IEI is recurring respiratory infections, which can both promote the development of bronchiectasis and be further aggravated by them. Most cases of symptomatic bronchiectasis in children and adolescents can be attributed to diseases with dominating lung involvement, e.g. cystic fibrosis and ciliary dyskinesia (38), a finding that is consistent with the statements of the pneumologists interviewed. Physicians reported that between 20% and 40% of cases remain unclarified (bronchiectasis of unclear origin or idiopathic bronchiectasis). All participants confirmed that in patients with bronchiectasis, IEI had been included in the differential diagnosis. In these cases, basic immunological diagnostics are usually performed in the pneumology clinics. However, six out of eight pneumologists stated that they consult immunology experts in-house or externally (see also: Diagnostic procedures and collaboration with specialized IEI centers).

#### Lymphoma predisposition in IEI patients

Lymphoma is one of the most serious complications of a number of IEIs, e.g. in autoimmune lymphoproliferative disease (ALPS) and APDS (13). Data suggest that exhaustion of CD8+ and possibly of CD4+ T cells significantly increases the risk of lymphoma (39, 40), as demonstrated in APDS (41). We therefore asked hemato-oncologists questions pertaining to IEI diagnosis in lymphoma patients with unusual findings, e.g. treatment intolerability or familial clustering of cases. Responses suggest that only few patients are diagnosed with IEI after being diagnosed with lymphoma, and most participants reported one case per year at most. Some of the participating hemato-oncologists reported that they paid particular attention to lymphoma patients with concomitant symptoms of autoimmunity or poor recovery after B-cell depletion by the administration of rituximab. Corroborating the literature, experience had shown that immune dysregulation more likely than susceptibility to infection is found in patients with IEI-associated lymphoma. The problem of treating patients with known IEI or syndromic diseases such as AT was reported several times, as these patients are usually more susceptible to therapy-related toxicity. In most cases, a curative approach is still taken and the dose is reduced or the treatment regimen is changed if necessary (42). In addition, patients may be given prophylactic antibiotics. In the case of AT, which can be caused by a variety of different mutations in the ATM gene, genetics can now be used in some cases to predict treatment tolerance (42).

#### Diagnostic procedures and collaboration with specialized IEI centers

With the increasing availability of effective treatment options for IEI, accurate diagnostics becomes more important. The participating pneumologists and hemato-oncologists were therefore asked which diagnostic procedures they perform to prove and specify suspected IEI cases and in what ways they collaborate with IEI centers.

The majority of the clinicians surveyed (5/8 pneumologists, 3/6 hemato-oncologists) stated that they were able to carry out a wide range of immunological examinations in their own clinic, including assessing lymphocyte subsets, complement system, antibody subclasses, and response to vaccination. For more extensive examinations, e.g. functional tests and genetics, however, patients were predominantly referred to specialized IEI centers.

### Treatment of IEI patients by clinic-based pneumologists and hemato-oncologists

Treatment options for PID patients can be divided into symptomatic (e.g., immunoglobulin replacement therapy = IRT, antibiotic administration), targeted and curative treatment (e.g., hematopoietic cell transplantation). The latter two treatment options are highly complex and require expertise for immunodeficient patients. We therefore asked our interview partners which treatment options they offer IEI patients and when a specialist center is consulted.

**Figure 6:**
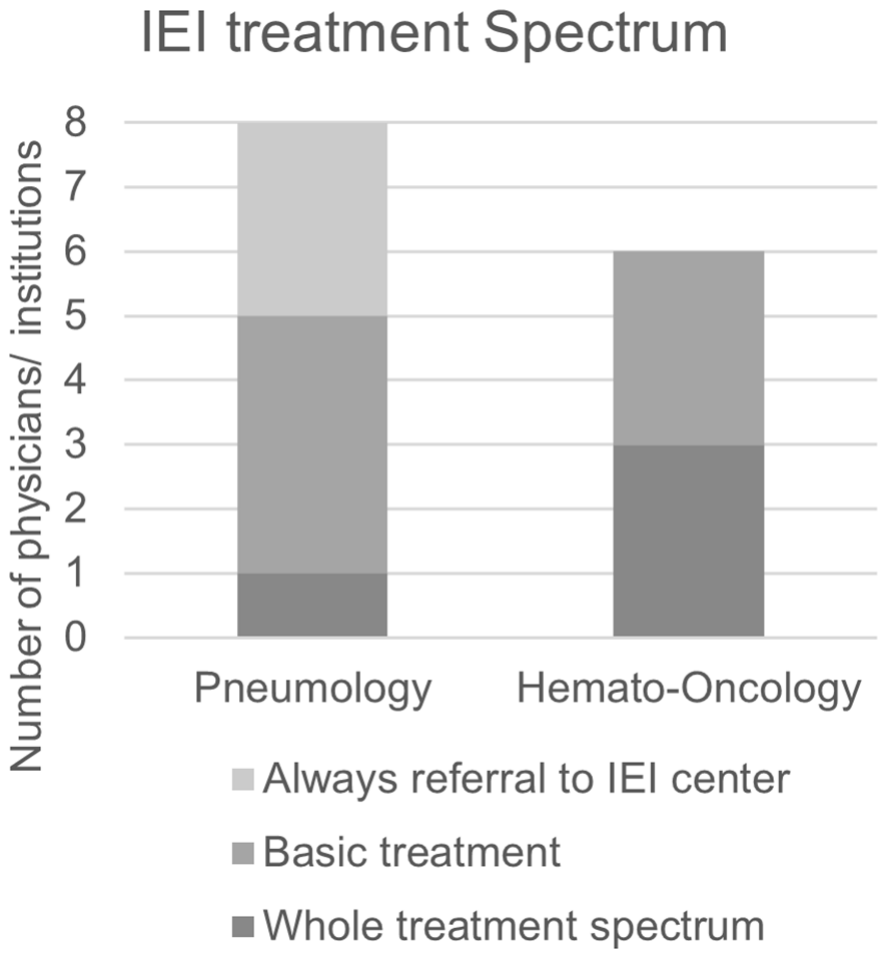
Treatment spectrum offered to patients with IEI by clinic-based pneumologists and hemato-oncologists. Always referral to IEI center: Clinic does not initiate or conduct treatment for IEI symptoms. Basic treatment: Clinic may conduct basic treatments including antibiotic prophylaxis and immunoglobulin replacement but will refer patients to a specialized IEI center for more advanced treatments. Whole treatment spectrum: Clinic offers all treatment options including targeted treatment.

Not surprisingly, all participants responded that organ-specific manifestations of IEI such as bronchiectasis and lymphoma are treated in their pneumology or hemato-oncology clinics. Furthermore, several participants from pneumology and hemato-oncology stated that they order and perform IRT themselves. In larger hemato-oncology clinics, there is often extensive expertise in HCT. However, several interviewees stated that they do not transplant IEI patients themselves or only in close coordination with an IEI center. This reflects the special characteristics of IEI patients compared to cancer patients in the transplant setting. For example, the decision whether or not to transplant requires specific knowledge on IEIs. There are also differences in the conditioning and care of IEI patients in the different phases after transplantation compared to cancer patients (29, 43). Targeted therapies have been used in hemato-oncology for some time, and with the advent of new drugs for the treatment of CF and autoinflammatory lung diseases, they are also becoming increasingly common in pneumology. However, participants from both pneumology and hemato-oncology predominantly stated that they do not carry out targeted therapies in IEI patients themselves but refer patients to a specialist center for this purpose (**Figure 6**). Participants who reported offering targeted therapies to IEI patients themselves were mostly those employed at a clinic specializing in IEI.

### Interest in immunological education: practice-based vs clinic-based physicians

**Figure 7:**
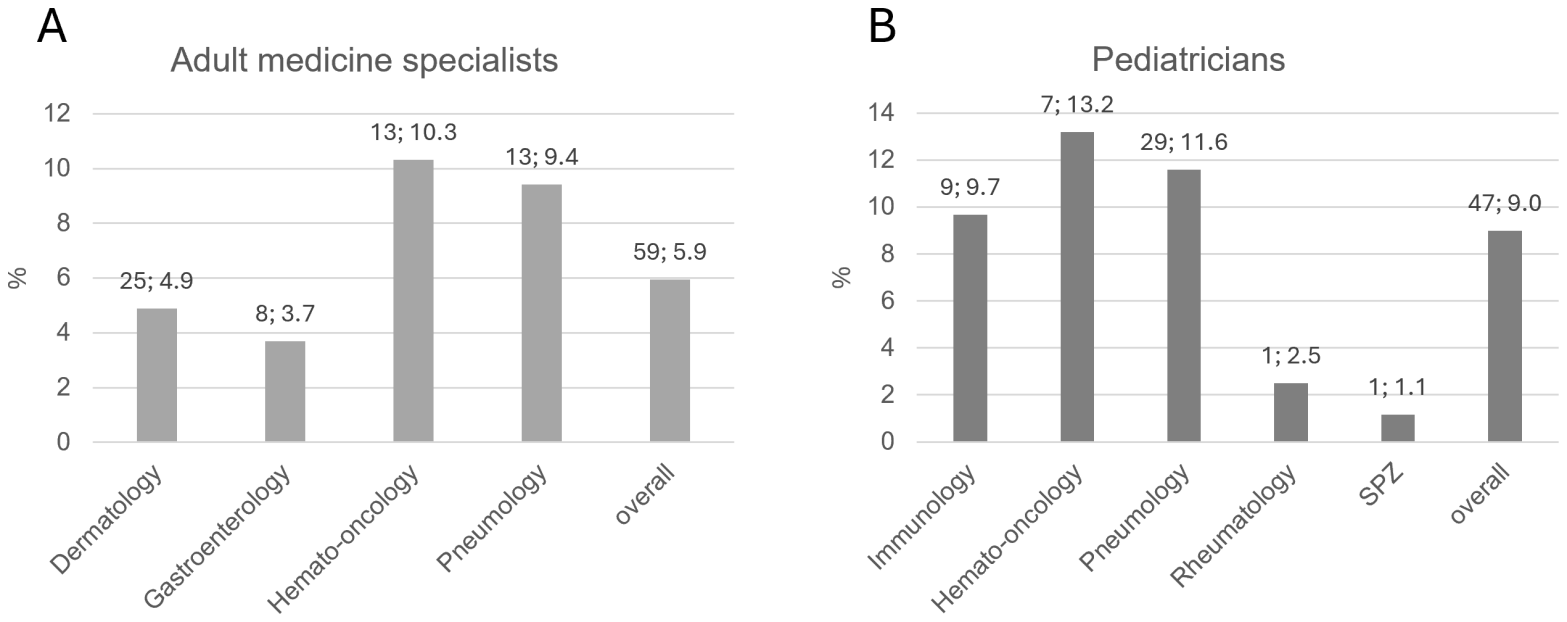
**(A)** Rate of practice-based physicians for adult medicine who were interested in receiving further information on IEI, by specialization. **(B)** Rate of practice-based pediatricians who were interested in receiving further information on IEI, by sub-specialization. Bars indicate percentages. Numbers above bar: absolute number; percentage. SPZ, social pediatric center.

A minority of practice-based physicians stated that further information on the topic of IEI was desired, and the responses demonstrated again that IEI is considered more important among pediatricians than adult physicians overall (adults: 59/992 = 5.9%; pediatricians: 47/523 = 9.0%). Differences were also seen between comparable sub-specializations (**Figure 7**).

Strikingly, the interest in educational material on IEI in each group of specialization or pediatric sub-specialization strongly correlated with the experience of having treated patients with IEI (**Supplementary Figure S1**).

In contrast to practice-based physicians, clinic-based pneumologists and hemato-oncologists had a high level of awareness and a significant knowledge of the topic. However, the comparison is limited by the facts that the surveys contained different questions and may also have a selection bias. All of the respondents of the clinic-based physicians considered further education on IEI to be very important (5/5 = very high priority) or important (4/5 = high priority). Several participants stated that they preferred training courses individually tailored to their need compared to more general events. For example, trainings highlighting the pneumological aspects of IEI were preferred to general introductions to IEI. Opinions differed on whether virtual events or on-site training should be preferred. Those in favor of virtual events pointed to practical considerations. Training sessions at the relevant specialist conferences were seen as a chance to reconcile the desire for personal exchange with the problem of potentially long journeys to in-person trainings.

## Conclusions, limitations and perspectives

The findings reveal a nuanced understanding of current knowledge and awareness, which leads to significant variation in the diagnosis and treatment of IEIs. Notably, there is substantial expertise and awareness of IEI within clinic-based pneumology and hemato-oncology centers across Germany. However, due to the self-selection bias it should be taken into account that physicians with an above-average expertise might have been overrepresented. Therefore, the same level of expertise or awareness cannot be assumed for all pneumology and hemato-oncology centers in Germany. But a remarkable deficit in knowledge in centers with a low expertise might be critical for the early detection and referral of suspected cases. Data suggests that pediatricians are generally better informed about IEI compared to adult physicians in this domain. This disparity likely stems from several factors. First, the majority of monogenetic diseases were impossible to diagnose prior to the advent of genetic testing. With the introduction of genetic testing and, more recently, neonatal screening, pediatricians have become the primary clinical point of contact. Additionally, many patients with severe IEIs historically succumbed to the condition during childhood, reducing adult physicians’ exposure to such cases.

As diagnostic and therapeutic strategies improve, coupled with the implementation of neonatal screening, patients with IEI are living longer. Consequently, it is increasingly important to enhance awareness among adult specialists to better identify the growing number of patients exhibiting clinical symptoms of IEI in distal organ systems, often linked to autoimmunity, beyond childhood. Furthermore, alongside childhood-onset IEIs caused by germline mutations, somatic mutations can lead to immune disorders that manifest in adulthood. Given that these patients are less likely to present with classic immunodeficiency, they are often undiagnosed during childhood, either by immunologists or geneticists (44).

Recent advancements have highlighted that a significant number of pathogenic genetic variants may manifest as immune system dysfunctions or dysregulations, necessitating enhanced interdisciplinary collaboration. This, in turn, opens up the potential for a unified diagnostic approach across a broad spectrum of diseases (5).

To achieve these objectives, targeted efforts are necessary to reach the appropriate medical groups. While those already sensitized to the issue recognize the importance of further education, it is critical that the educational content be directed towards the medical professionals who remain insufficiently informed. The relatively low to moderate interest in additional information on IEI, as indicated by telephone surveys with practice-based physicians, underscores the essential role of professional societies in outreach initiatives. These organizations and other trusted institutions can provide effective communication channels that resonate with the target audience. For practice-based physicians, foundational symposia featuring illustrative case studies could prove beneficial in raising awareness, though it is crucial to consider that IEIs are rare diseases, and the complex symptom spectrum is challenging to convey in isolated events.

The low participation rates, regional imbalance and potential self-selection bias in the surveys warrant cautious interpretation of the results. Although contact by telephone was quite successful in the case of practice-based physicians, it proved to be less effective for clinicians, possibly because clinics are often overburdened, and the lack of contact persons. Increasing the participation rate is a challenge. One approach could be a cooperation with relevant specialist societies, which might significantly increase the participation rate and might also improve regional balance to enhance inclusion of participants in the Northern and Eastern parts of Germany and potentially additionally in other regions of Europe. Stratified sampling along factors like size of institution or publication history of clinicians could be used to avoid the skewing of results by self-selection.

In this study there is a lack of correlation between the knowledge and diagnostic practices of the physicians surveyed and specific clinical patient outcomes, such as the time to diagnosis, treatment success, or progression. Future studies could specifically supplement these aspects by incorporating registry data or clinical follow-up data in order to better quantify and document the influence of physician awareness on the reality of care for patients with IEI.

In summary, the results indicate a complex and varied perception of IEI in the wider medical community. Going forward, it is crucial to raise awareness and improve education regarding IEI, particularly for physicians who mainly treat adults, to better address its increasing prevalence and clinical complexity.

## Data Availability

The original contributions presented in the study are included in the article/**Supplementary Material**. Further inquiries can be directed to the corresponding author.
