# Correction: A survey of diagnosis and therapy of inborn errors of immunity among practice-based physicians and clinic-based pneumologists and hemato-oncologists

**DOI:** 10.3389/fimmu.2025.1666329

**Published:** 2025-08-07

**Authors:** Thomas Lehrnbecher, Alexandra Russo, Gernot Rohde

**Affiliations:** ^1^ Department of Pediatrics, Division of Hematology, Oncology and Hemostaseology, Johann Wolfgang Goethe University, Frankfurt/Main, Germany; ^2^ Department of Pediatric Hematology/Oncology, Center for Pediatric and Adolescent Medicine, University Medical Center, Johannes Gutenberg-University, Mainz, Germany; ^3^ Department of Pneumology, Clinic of Pneumology, Intensive Care Medicine and Sleep Medicine, University Medical Center, Justus Liebig-University, Marburg, Germany

**Keywords:** IEI, PID, APDs, immunodeficiency, survey, diagnostic landscape, treatment landscape, health services research

In the published article, there was an error in **Supplementary Table 1**, Questionnaire 2 Answers HaemO. The correct file has now been published.

The original version of this article has been updated.

