# Supplementary material for: A survey of diagnosis and therapy of inborn errors of immunity among practice-based physicians and clinic-based pneumologists and hemato-oncologists
Source: Front Immunol. 2025 Jun 5;16:1597635. doi: 10.3389/fimmu.2025.1597635 (PMC12177555; doi:10.3389/fimmu.2025.1597635)
Supplement: Supplementary file 3 [file DataSheet1.docx]

Supplementary material

Supplementary figures

| 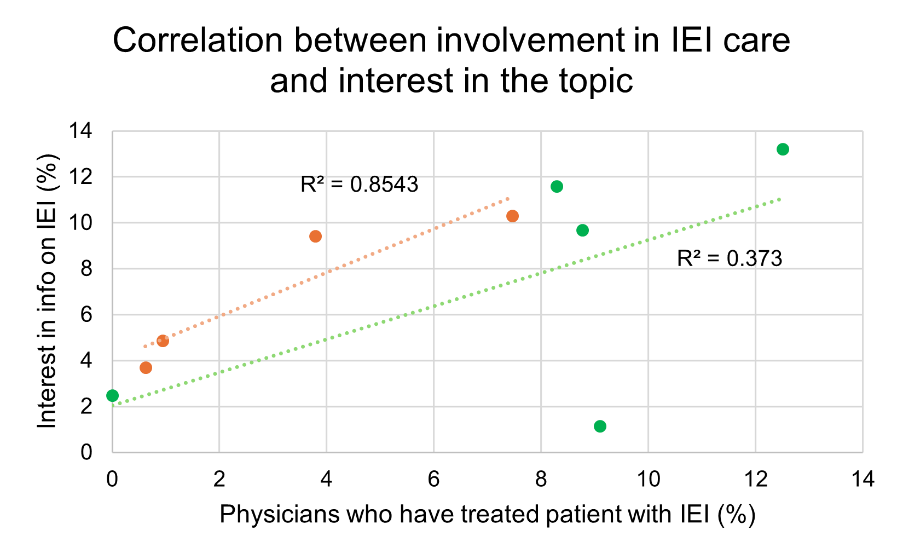 |
| --- |
| **Figure S1:** Correlation between involvement in IEI care and interest in IEI educational material by specialization. Orange dots represent specializations in adult medicine; green dots represent sub-specializations in pediatric medicine. |

Questionnaires clinic-based specialists

**Questionnaire 2: Clinic-based pneumologists.** Questionnaire with a common set covering questions on the participants’ institutions and experience with IEI, general IEI diagnostics and IEI-focused trainings. In addition, a part specific to pneumology, covering disease manifestations and diagnostic procedures specific to pneumology.

1. What is the size of your facility (beds, new admissions per year)?

2. How important is the topic of PID within your patient population (number of suspected PID cases, number of confirmed cases, number of PID patients treated per year)?

3. How many years of professional experience do you have a) in total b) as a specialist c) as a specialist in pneumology?

4. How often do you see bronchiectases of unclear genesis?

5. If the cause is unclear, is a PID considered as the cause?

a) If “Yes”: is (a) a genetic test carried out in your institution or (b) referred to an immunologist?

b) If “No”: what would be the next steps?

6. How many patients (children/adults) with non-CF bronchiectases are currently being treated at your institution, how many of them specifically with “unclear genesis”?

7. Is your facility recommended as a point of contact for PID (e.g. by patient organizations)?

a) If no: Are PID patients referred back to you from the PID centers?

I. If this is the case: when and with what question?

8. In which situations do you establish a strong, slight or no suspicion of PID

a) Anamnestic and/or clinical evidence of increased susceptibility to infection (e.g. chronic purulent bronchitis, chronic productive cough, chronic bacterial sinusitis, chronic lymphadenopathy, recurrent fever) or immune dysregulation

b) Abnormal laboratory findings (immunophenotype)

c) Certain organ manifestations (e.g. bronchiectases)

d) Developmental disorders in children?

9. Do you know or use the recommendations of the “European Society for Immunodeficiency” or the AWMF guidelines “ELVIS” and “GARFIELD” to screen for immunodeficiencies?

10. If a PID is suspected, are immunological tests carried out at your institution?

a) If yes, which of the following diagnostic steps do you carry out in-house?

- Differential blood count incl. (I) determination of immunoglobulin levels (II) T- and B-cell FACS analysis (III) vaccination antibodies, e.g. against tetanus and pneumococci

- Immunophenotyping, if necessary, with lymphocyte function tests

- Genetic testing: if yes, performed internally or externally (where?)

b) Which of the aforementioned tests have you requested yourself in the last 12 months?

11. What experience do you have with the prescription of orphan drugs or high-priced drugs?

12. If a patient is found to have a PID as an incidental finding, would you initiate targeted therapy yourself or refer them to a PID center?

13. Further training on the subject of PID

a) How important are further training courses on the subject of PID for you (1-5; 1 = not important, 5 = very important)?

b) Which questions would you like to see addressed there and which format (in person, virtual...) do you prefer?

c) How many immunology training courses have you already attended?

**Questionnaire 3: Clinic-based hemato-oncologists.** Questionnaire with a common set covering questions on the participants’ institutions and experience with IEI, general IEI diagnostics and IEI-focused trainings. In addition, a part specific to hemato-oncology, covering disease manifestations and diagnostic procedures specific to hemato-oncology.

1. What is the size of your facility (beds, new admissions per year)?

2. How important is the topic of PID within your patient population (number of suspected PID cases, number of confirmed cases, number of PID patients treated per year)?

3. Does your institution perform stem cell transplants?

4. How many years of professional experience do you have a) in total b) as a specialist c) as a specialist in hemato-oncology?

5. How often do you see patients with lymphoma who are younger than the expected age of onset of the respective lymphoma, e.g. DLBCL?

6. Have you seen patients with suspected lymphoma who ultimately turned out to be PID patients?

7. Is the possibility of primary immunodeficiency also considered as a cause when diagnosing lymphoma, especially in young patients? If so, when?

8. What diagnostics do you carry out if PID is suspected as a comorbidity, or do you then refer to a PID expert (which center)? What is or is not tested and why?

9. How does the treatment regimen change if PID a) is known or b) is diagnosed during the course of the disease?

10. If PID is only diagnosed after oncological therapy has started, does this have an influence on the success of the oncological therapy in your experience?

11. Is your institution recommended as a contact point for PID (e.g. by patient organizations)?

a) If no: Are PID patients referred back to you from the PID centers?

I. If no: Is there a PID center in your region to which you usually refer patients?

12. In general: What symptoms make you think of PID?

a) Anamnestic and/or clinical evidence of increased susceptibility to infection + immune dysregulation

b) Abnormal laboratory findings (immunophenotype)

c) Benign lymphoproliferation (e.g. lymphadenopathy, organomegaly)

d) Developmental disorders in children?

13. Do you know or use the recommendations of the “European Society for Immunodeficiency” or the AWMF guidelines “ELVIS” and “GARFIELD” to screen for immunodeficiencies?

14. If a PID is suspected, are immunological tests carried out in your institution?

a) If yes, which of the following diagnostic steps do you carry out in-house?

- Differential blood count incl. (I) Determination of immunoglobulin levels (II) T- and B-cell FACS analysis (III) Vaccine antibodies

- Immunophenotyping, if necessary, with lymphocyte function tests

- Genetic testing: if yes, internal or external (where?)

b) Which of these have you requested yourself in the last 12 months?

15. If a patient is found to have a PID as an incidental finding, would you initiate targeted therapy yourself or refer the patient to a PID center?

16. Further training on the subject of PID

a) How important are further training courses on the subject of PGD for you (1-5; 1 = not important, 5 = very important)?

b) Which questions would you like to see addressed there and which format (in person, virtual...) do you prefer?

c) How many immunology training courses have you already attended?
